# Comparative study of conventional frying and air frying on the quality of potatoes (*Solanum tuberosum* L.)

**DOI:** 10.1002/fsn3.3617

**Published:** 2023-08-09

**Authors:** Jonathan Coria‐Hernández, José Luis Arjona‐Román, Rosalía Meléndez‐Pérez

**Affiliations:** ^1^ Laboratory 13 Thermal and Structural Analysis of Materials and Foods National Autonomous University of Mexico‐Superior Studies Faculty at Cuautitlan (UNAM–FESC) Campus 4 Multidisciplinary Research Unit Cuautitlan Izcalli Mexico

**Keywords:** air frying, conventional frying, food quality, French fries, potato

## Abstract

The human being has historically consumed fried foods for centuries; however, conventional frying has a disadvantage, immersion in vegetable and/or animal oils, which leads to the search for different options. This is why air frying is a good alternative, which still has a wide field of study. In this work, frozen French fries of a brand marketed in Mexico that were subjected to frying in canola oil and air frying were compared. They were evaluated through the change in the removed moisture content, water activity, color profile, hardness, fracturability, and surface damage by SEM, thermal analysis by MDSC, and chemical by FTIR‐ATR spectroscopy. Air‐fried French fries were found to contain about 48% less moisture, fewer perceptible color changes, and less surface damage translated into better crunchiness compared with conventionally fried. It was also found that the changes at the chemical level are smaller, mainly attributed to the absence of canola oil and that the thermal transitions are more stable in terms of temperatures and enthalpies, which makes it possible to emphasize that air frying is a good alternative for developing new fried products that allow expanding the variety of these in the market without sacrificing some quality attributes.

## Graphical abstract

Comparison of conventional immersion frying and air frying. Potato quality subjected to two frying processes. Thermal and structural analysis of French fries.

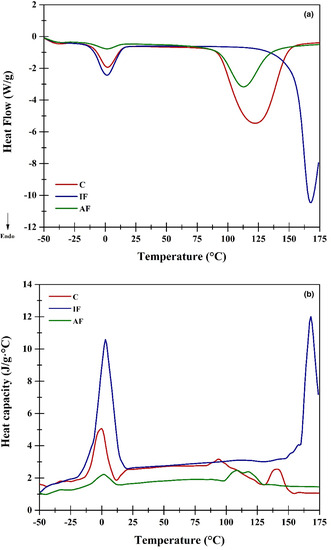

## 1 INTRODUCTION

Over the last few decades, the production and consumption of French fries have increased exponentially, with various implications such as a processing and sourcing operation that has several carefully controlled steps to transform raw potatoes into fries with a crispy crust and deep golden‐brown color appetizing and sought after by consumers (Leong et al., 2022).

Potatoes (*Solanum tuberosum* L.) are classified as one of the foods rich in nutrients, which in turn have good potential to alleviate nutritional deficiencies in the human diet. However, the chemical changes that are associated with frying end up compromising its integrity in terms of sensory and nutritional quality. During the manufacturing process, a series of chemical reactions occur, which are primarily associated with changes in the moisture and oil content of the potatoes (Ngobese & Workneh, 2018).

In general, it is found that potatoes contain a complex carbohydrate known as starch, which constitutes most of the total dry matter of the tuber (between 60% and 80%), so it is important to consider that in industrial applications, serves as an indicator of the final quality of the fries in terms of texture and color.

It has been found that the consumption of French fries is common and popular in homes and food service establishments, which causes its long‐term consumption to be closely related to health problems, such as cardiometabolic diseases, type 2 diabetes, and hypertension. Therefore, it is necessary to develop alternatives that allow careful consideration of nutritional quality aspects without affecting sensory quality, using processing technologies that are adapted to the commercial manufacture of French fries (Leong et al., 2022).

Acrylamide is a compound that belongs to the group of the most powerful carcinogens and neurotoxins that can be found in foods, such as French fries, potato chips, coffee, among others, which is a byproduct of Maillard reactions between L‐asparagine with various reducing sugars when heated above 120°C for at least 20 min continuous (Pantalone et al., 2023). Due to their broad characteristics of toxicity and carcinogenicity, acrylamides have been classified by the World Health Organization as a Group 2A carcinogen, especially genotoxic (Başaran & Turk, 2021). French fries are the food products most susceptible to the formation of acrylamide due to their higher content of L‐asparagine and reducing sugars, in addition to the traditional conditions of frying by immersion in oil (heating that can exceed 120°C). Several types of food have been reported to have acrylamide levels, especially French fries, and various thermally processed products, which is why acrylamide has not been detected in unheated raw foods, ensuring that the role played by overheating is important for the formation of acrylamides (Yassin et al., 2022).

Frying is conventionally used as a domestic and commercial cooking method to transform and/or preserve foods with distinctive sensory characteristics. This process, also known as ambient pressure frying or immersion frying, consists of immersing food in vegetable or animal oils at temperatures ranging between 120°C and 200°C (Del Rosario‐Santiago et al., 2022). These conditions allow the generation of physical and chemical changes, such as starch gelatinization, surface crusting, formation of flavor components, shrinkage, and swelling. These alterations result in modifications at the macro‐ and microstructural levels. Various investigations in this area suggest that one of the critical problems with fried foods is the relatively high‐fat content, which has been closely linked to a significant increase in the number of cardiovascular diseases and cancers (Al Faruq et al., 2022; van der Sman & van den Oudenhoven, 2023).

Air frying is a recently incorporated and innovative method for preparing fried foods and is considered an alternative to the conventional frying process since it produces fried foods with very little or no oil content. Generally, food products are fried with forced circulation of hot air instead of immersion in oil, and the frying chamber radiates dry heat from the source for more effective and faster frying, which is reflected in the formation of crusts like those obtained by the traditional process (Heredia et al., 2014).

One of the main phenomena that occur in air frying is that food is processed by direct interaction between the external emulsion of oil droplets in hot air and the food in the frying chamber. The heat transfer rate in this process is remarkably high and is evenly distributed throughout the food, resulting in high‐quality fried products (Ran et al., 2023).

Another advantage of this process is that the amount of oil required to be used is considerably less (approximately 50%–70% less) compared with the conventional process, which results in foods with low fat content, significantly modifying the color, texture, flavor, and aroma (Dehghannya & Ngadi, 2021).

Various studies have shown that lipid oxidation and the Maillard reaction are the most common chemical reactions involved in the development of fried flavor; thus, the oil content can alter several sensory characteristics of the final product. Since air frying can be carried out using less oil, fat absorption, and degradation problems are becoming less important and could reduce the formation of acrylamides compared to deep frying.

Mesias et al. (2021a); Mesias et al. (2021b), and Sansano et al. (2015) stated that air frying decreased the acrylamide content by approximately 90% compared with the traditional process. However, relatively limited studies are currently available to explain the mechanisms and kinetics of mass transfer during the air frying process (Ran et al., 2023). In addition, air frying technology can produce foods with low oil content, which allows for generating a wide field of research on the physical and physicochemical characteristics of foods fried by this process. That is, as a result, more research is required to develop knowledge and understanding of this technique and expand its application to homes and industries (Al Faruq et al., 2022; Yu et al., 2020).

This research aims to study the physicochemical and quality changes that occur when comparing two frying methods: (1) the conventional one, by immersion in canola oil, and (2) the forced air convection frying, on some of the quality attributes of French fries and thereby generate new information that allows expanding and diversifying the use of this novel frying technology.

## 2 MATERIALS AND METHODS

### 2.1 Sample preparation and processing conditions

Frozen potatoes (*Solanum tuberosum* L.) of a commercially recognized brand in Mexico were used, which were kept under storage conditions in a forced convection chamber (Torrey) at −18°C ± 1°C, which belonged to the same production lot, and they were classified according to their dimensions of 60 × 10 × 9 mm (length × width × thickness), and thus avoid systematic errors in the research.

Two methods were used for the potato frying process: (1) conventional by immersion in canola oil, which was carried out in a 10‐inch‐diameter stainless steel wok, and (2) air frying by forced convection, using an Actifry (T‐fal, SEB) with the frying chamber previously preheated to 200°C for 3 min, air speed of 2.85 m/s, and rotation speed of the internal paddle of 30 rpm. In both cases, 100 g of frozen potatoes were used, which were fried at 180°C ± 2°C for 8 min.

The samples analyzed were classified as follows: *C*: control samples fresh from the container without frying treatment; *IF*: samples treated by immersion frying, and AF: samples treated by air frying.

### 2.2 Moisture content and water activity (*a*_w_)

The moisture content (%) of the samples was determined according to the methodology described by the A.O.A.C. (2005), by drying them in a hot air oven (Figursa) at 70°C ± 1.5°C for 24 h until a constant weight was achieved in five replicates.

The *a*_w_ value was determined according to the methodology described by van der Sman (2023) with a 4TE dew point hygrometer (Aqualab) at 25°C ± 1.5°C with five replicates.

### 2.3 Color profile parameters

Tri‐stimulus values (L* = lightness, a* = redness, and b* = yellowness), and total color changes (∆E*) were determined in the five samples per treatment. The technique used was reflectance spectrophotometry according to the CIE system, using a Miniscan EZ 4500 spectrophotometer (Hunterlab) with type D_65_ illuminant (daylight at 6504 K), sphere opening of 8 mm and 10° viewing angle (Mesias et al., 2021b).

### 2.4 Texture analysis

A CT3 texture analyzer (Brookfield) with a 3 mm flat cutting blade (SB geometry) was used at a test speed of 1 mm/s for 20 s and an activation load of 0.05 kg_f_, the cross‐section was performed at 23°C ± 1°C with five replicates per treatment. The parameters considered for the analysis were the maximum force represented as hardness (kg_f_) and fracturability (kg_f_) representing the crunchiness (Sadeghi et al., 2021).

### 2.5 SEM analysis

The surface morphology of the French fries was dehydrated and de‐oiled was observed using a JSM‐6010LA microscope (JEOL) at 200×. Samples were gold coated by sputtering at 10 mA for 10 min in a Denton Desk V ion sprayer (Denton Vacuum) to improve image quality (Zhang et al., 2023).

### 2.6 Modulated differential scanning calorimetry (MDSC)

Five replicates per treatment of French fries samples (6.0 ± 0.2 mg) were thermally analyzed using a 2920 Temperature Modulated Differential Scanning Calorimeter (TA Instruments), calibrated at baseline and cell constant with Indium (156.6°C) and heat capacity with sapphire at 5°C/min. Nitrogen was used as the purge gas at a constant flow of 60 mL/min with a Refrigeration Cooling System (RCS). The test was carried out from −50°C to 175°C, modulated to ±0.796°C every 60 s. The obtained thermograms were analyzed using TA Instruments Universal Analysis software, 2000 V 4.5 A.

### 2.7 Fourier transform infrared spectroscopy with attenuated Total reflection (FTIR‐ATR)

A Frontier SP8000 spectrophotometer (Perkin Elmer) accessorized with an in‐compartment diamond ATR accessory was used to characterize the specific functional groups present in the samples. Five samples per treatment were placed and measured in transmittance mode after pressing them on the ATR crystal. Infrared reflection was evaluated in the wavenumber range of 4000 to 450 cm^−1^ at a resolution of 4 cm^−1^ by co‐adding 32 scans.

### 2.8 Experimental design and statistical analysis

The experiment was carried out as a completely randomized design with five replicates. Mean, standard deviation, 1‐way and 2‐way ANOVA, and comparison of means by the Tukey test were performed using the Minitab 16.0.1 software (Penn State University). A significance value of *p* < .05 was used to identify significant differences between treatments.

## 3 RESULTS AND DISCUSSION

### 3.1 Moisture content and *a*_w_

Moisture content is an important parameter in products that are processed by frying, since being an operation studied from the perspective of heat and mass transfer, it is relevant to consider the changes that occur with potatoes. Table 1 presents the results related in the first instance, to the percentage of weight loss, where it is observed that there are significant differences (*p* < .05) between both frying methods. It was found that immersion frying (*IF*) does not remove enough water from the samples, this is mainly attributed to the transfer phenomena (heat and mass) that occur by immersion in oil, causing thicker crusts and therefore, affecting the migration of water from the interior to the exterior, as mentioned by Gouyo et al. (2021) and Ran et al. (2023).

**TABLE 1:** Results of moisture, water activity, color profile, and texture of the analyzed French fries samples.

	Sample		
	*C*	*IF*	*AF*
Weight loss (%)	–	36.55 ± 1.05^a^	53.40 ± 0.92^b^
Moisture (%)	78.86 ± 0.91^c^	65.76 ± 1.98^b^	40.67 ± 1.16^a^
*a*_w_	0.96 ± 0.01^b^	0.96 ± 0.01^b^	0.91 ± 0.01^a^
L*	95.51 ± 0.98^b^	87.96 ± 1.00^a^	96.18 ± 0.51^b^
a*	−8.79 ± 0.24^b^	−2.57 ± 0.21^a^	−7.86 ± 0.11^b^
b*	42.74 ± 1.80^a^	60.05 ± 1.26^b^	47.19 ± 0.96^a^
∆E*	–	5.14 ± 0.27^b^	3.43 ± 0.02^a^
Hardness (kg_f_)	0.08 ± 0.00^a^	2.21 ± 0.48^b^	3.05 ± 0.12^c^
Fracturability (kg_f_)	0.04 ± 0.00^a^	0.04 ± 0.01^a^	0.07 ± 0.00^b^

*Note*: Mean ± Standard deviation. Means with different letter in the same row, are statistically different (*p* < .05).

Concerning the moisture values, it was found that there are differences (*p* < .05) between the samples analyzed, finding that there is a close relationship between this parameter and weight loss, with the *AF* samples being the ones that eliminated the most water, results that agree with those presented by Ran et al. (2023), since the forced convection of air inside the fryer causes the water to be removed at lower rates, causing the formation of crusts and starch gelatinization not to occur as quickly, allowing the migration of internal water.

The water activity value allows us to know the status of the free water in the samples, in the case of foods that go through water removal processes, this physicochemical property is extremely important since it was found in Table 1 that even though the moisture values are different between the processes, the *C* and *IF* samples do not present differences (*p* > .05). This phenomenon is mainly attributed to the fact that in sample *AF*, the water that remained inside the French fries modified their interactions at the molecular level, allowing interactions to be generated mainly with starch, minimizing its mobility, unlike samples *C* and *IF*, which agrees with the results obtained by the MDSC thermal and molecular FTIR analysis (van der Sman, 2023; Verma et al., 2023).

### 3.2 Color profile parameters

The frying process generates important changes at a superficial level in the materials; the case of French fries is not isolated from these optical and chemical phenomena. The color of French fries is the result of the chemical caramelization reaction (Maillard) that depends on the surface content of reducing sugars, the temperature, and the frying time (Moyano & González, 2002). Table 1 shows the results obtained from the tri‐stimulus coordinates in terms of color by reflectance. For the luminosity coordinate (L*), it was found that there are no differences (*p* > .05) between the *C* and *AF* samples, indicating that air frying does not generate apparent changes, unlike the *IF* samples, which their value of L* decreases significantly (*p* < .05), that is, a parent is darkening of the samples due to the Maillard reactions and which are by those presented by Iglesias‐Carres et al. (2023).

The a* coordinate (redness‐greenness), it was found that again the *IF* sample is different (*p* < .05) from the other treatments (*C* and *AF*); however, it is important to consider that the browning of the French fries not only it depends on the L* coordinate, if not on a combination of the three coordinates, providing the characteristic color of this type of food. Likewise, b* (yellowness‐blueness) obtained similar results regarding the treatments, agreeing with the results presented by Mesias et al. (2021b).

Significant differences (*p* < .05) were found in the total color changes (∆E*) between the *IF* and *AF* samples, taking sample *C* as a reference. This is an expected behavior, given the marked differences found in the evaluated tri‐stimulus coordinates. In both cases it was found that they are greater than 1, indicating that they are perceptible changes to the human eye and that they strongly influence the perception of consumers. However, it is important to highlight that although the samples are different, the treatment with *AF* has a lower value, indicating that it is the one that would affect the perception of color to the consumers to a lesser extent.

### 3.3 Texture analysis (hardness and fracturability)

The hardness was represented as the maximum force to cut the French fries, in this case, Table 1 shows that the hardness increases significantly (*p* < .05) regardless of the frying process (*IF* or *AF*) that is chosen. However, it is important to mention that in this case, the *IF* process causes the samples to have less hardness compared with the *AF* samples, because immersion in oil causes counter‐diffusion phenomena between the internal water of the potatoes and the oil generating an increase in the oil content, which generates a kind of internal and external coating in the samples, resulting in greater flexibility at the structural level and therefore less resistance to cutting, as observed in the SEM micrographs. The phenomenon was different from that which occurs with air frying, where surface water evaporates, causing the structure of the French fries to become more friable and harder (Gouyo et al., 2020; Sadeghi et al., 2021).

Gouyo et al. (2020) and Lisińska et al. (2007) have studied the crunchiness of French fries using mechanical tests (mainly through fracturability). Showing that during a texture analysis by shear force tests, a constant and defined force can be exerted on a fried sample when a blade is moved through it that mimics a first bite of the product. Subsequently, the force‐deformation curve is used to calculate the quantitative parameters related to the brittleness of the crust. Table 1 shows that the fracturability of the *C* and *IF* samples are the same (*p* > .05) because the oil plays a coating role on the potatoes, which is reflected not only in the hardness but also in the crunchiness; unlike the *AF* samples, where this value increases (*p* < .05), since the appreciation of the texture is a determining factor in consumer acceptance, in this case, that crunchiness value in the *AF* samples is beneficial for this type of product.

### 3.4 SEM analysis

**FIGURE 1:**
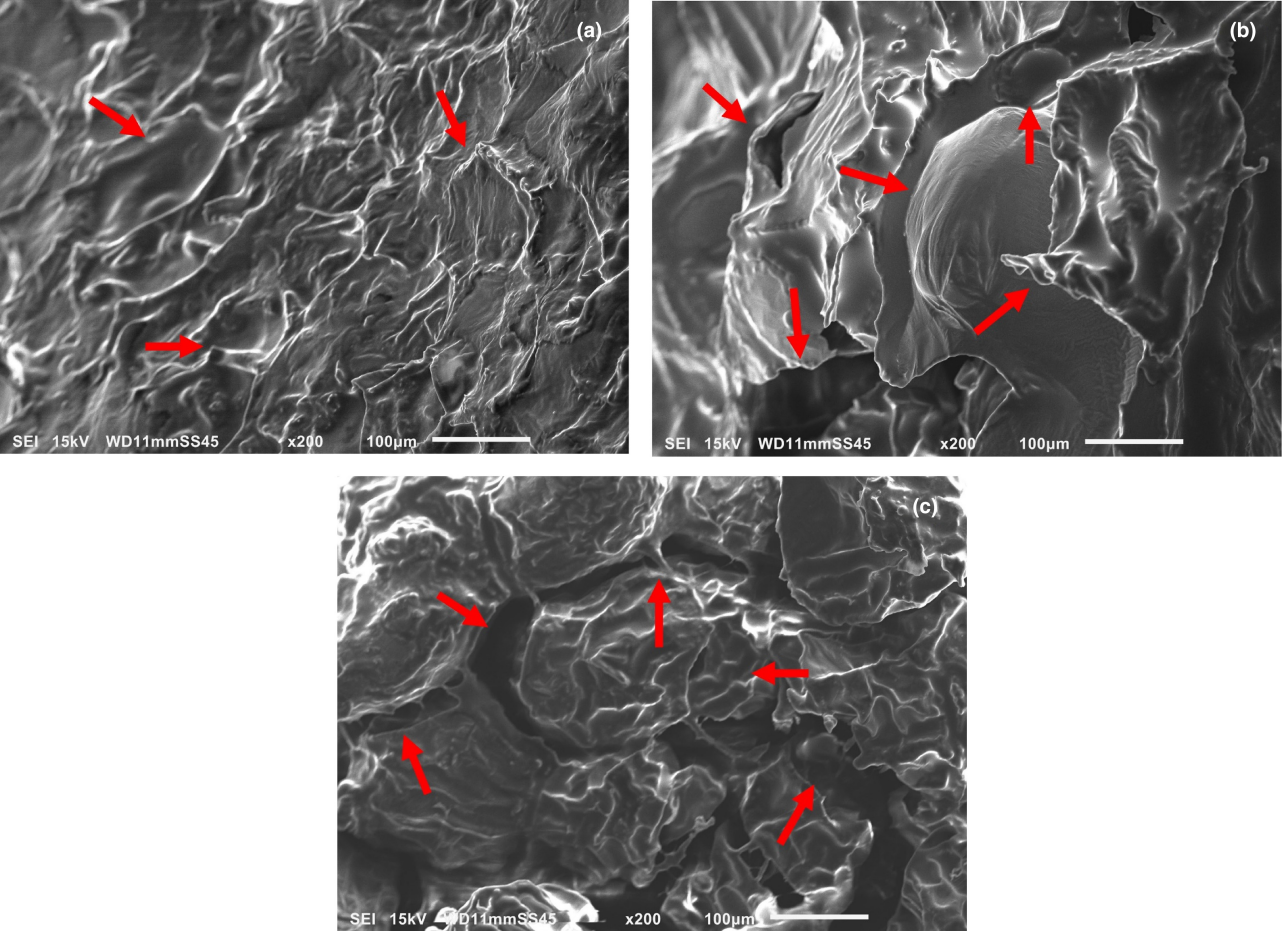
SEM micrographs at 200× of the samples analyzed (a) Control; (b) sample IF and, (c) sample AF.

Figure 1 shows the SEM micrographs of the samples. In the case of Figure 1a, we have the control of French fries (*C*), where it was found that at the surface level, there are no important changes in their structure since the starch granules maintain the surface of the fries without cracks and uniform samples that have not been treated by any frying method, results that agree with those presented by Li et al. (2022).

Figure 1b shows the French fries that have been processed by immersion, where, unlike sample *C*, significant superficial damage is already appreciated, this is mainly because the oil causes changes in the starch of the samples, generating ruptures and making the surface rough and rough, which is related to the values obtained in hardness and fracturability (Li et al., 2020).

Figure 1c corresponds to the *AF* samples, where a more uniform surface can be seen compared to the *IF* samples, and although cracks were found, they are slightly joined together, forming firm agglomerates that hold the structure together, maintaining mostly its integrity.

### 3.5 Thermal analysis by MDSC

**FIGURE 2:**
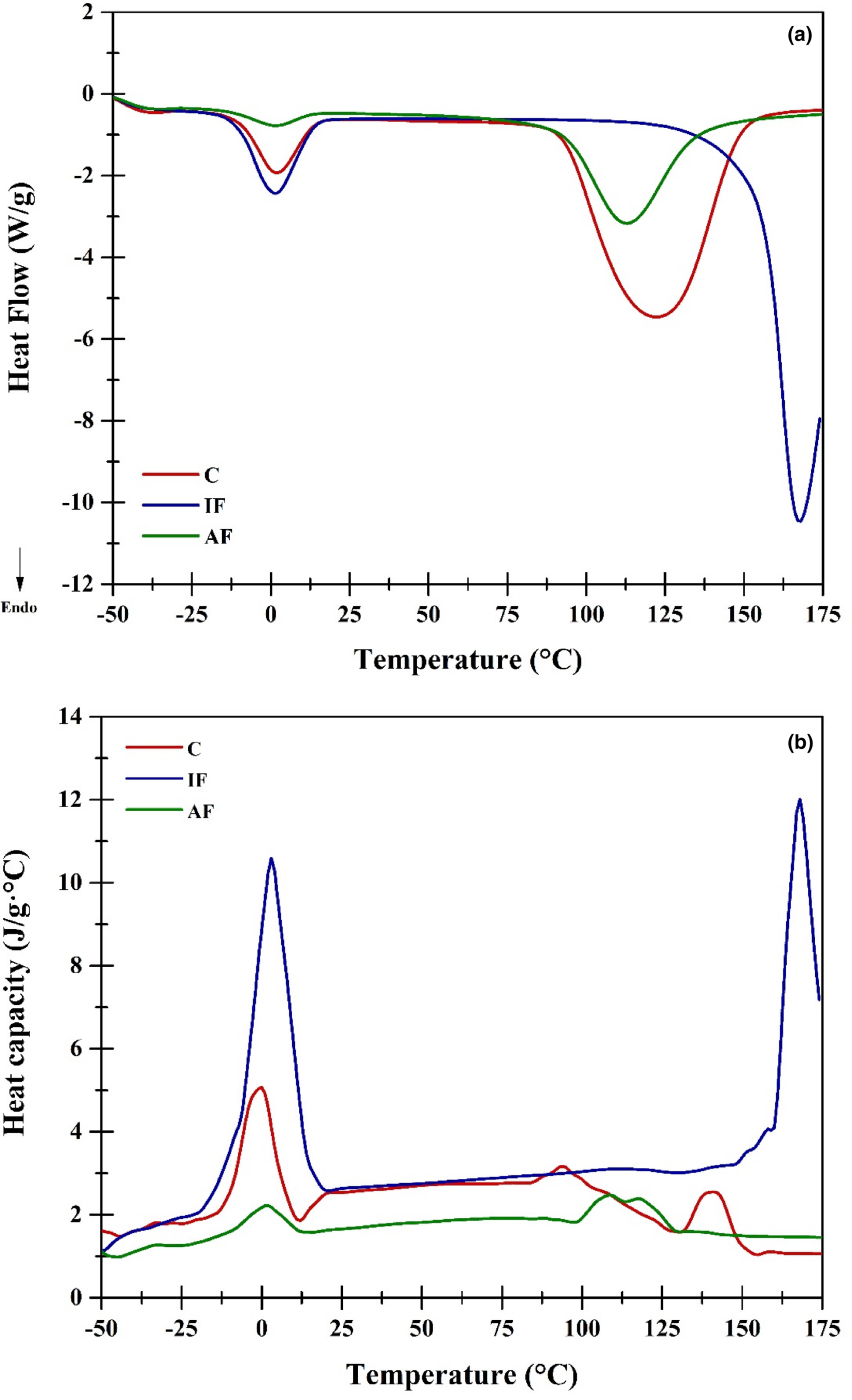
Thermograms obtained by MDSC of the French fries to the different treatments (a) Total heat flow (W/g) and, (b) Heat capacity‐Cp (J/g°C).

Thermal analysis by MDSC showed that there are three important transitions observed in the Total heat flow (Figure 2a). The first transition corresponds to the melting of the ice crystals of the samples, where it was found that the *C* and *IF* samples do not have differences (*p* > .05) in this zone as shown in the results of Table 2. This could be caused because the changes in the rearrangement of the internal water of the samples cause the energy values to be very similar, a behavior reported by Li et al. (2022).

**TABLE 2:** Results of thermal analysis by MDSC for all samples of French fries.

Zone	Sample		
	*C*	*IF*	*AF*
Melting			
Onset (°C)	−9.99 ± 0.02^a^	−10.78 ± 0.12^a^	−13.23 ± 0.05^b^
Maximum (°C)	1.71 ± 0.01^a^	1.44 ± 0.09^a^	1.34 ± 0.04^a^
Enthalpy (J/g)	127.10 ± 2.61^b^	180.30 ± 6.41^b^	33.87 ± 1.02^a^
Starch gelatinization			
Onset (°C)	37.30 ± 0.15^a^	51.36 ± 0.36^b^	40.98 ± 0.64^a^
Maximum (°C)	49.49 ± 1.02^a^	62.89 ± 2.21^b^	45.63 ± 0.92^a^
Enthalpy (J/g)	0.96 ± 0.01^b^	0.04 ± 0.00^a^	0.54 ± 0.01^b^
Evaporation			
Onset (°C)	92.54 ± 1.03^a^	100.41 ± 1.96^b^	92.27 ± 0.98^a^
Maximum (°C)	122.62 ± 0.64^a^	148.36 ± 2.66^b^	112.94 ± 1.01^a^
Enthalpy (J/g)	587.90 ± 1.95^a^	1085.00 ± 3.39^b^	446.52 ± 1.17^a^

*Note*: Mean ± standard deviation. Means with different letter in the same row, are statistically different (*p* < .05).

For the samples subjected to air frying (*AF*), it was found that the melting transition is executed at a maximum point equal (*p* > .05) to that of the *C* and *IF* samples; however, with an item of much lower energy expenditure, this is attributed to the moisture value of these samples being very low and this causes a smaller amount of frozen water, and therefore, it costs less work to melt a smaller amount of ice crystals (Allan et al., 2023).

The second important transition found in the MDSC analysis was between 15°C and 85°C, which is the one that corresponds mainly to the area where starch gelatinization occurs. In this sense, Table 2 shows the results obtained for the transition, which agree with those reported by Yang et al. (2020). These authors indicate that the values of starch gelatinization temperatures are modified if there is or is not the presence of oil during immersion frying, indicating that there are differences (*p* < .05) between samples *C* and *IF*. It is important to mention that they also found that the ΔH values are closely related to the amount and chemical state of the starch, indicating that the lower the enthalpy value, the lower the amount of starch in its native state.

It was found that for the *IF* samples, the enthalpy value is lower (*p* < .05) compared with the *C* and *AF* samples, indicating that the process structurally modified the starch, which is consistent with the results obtained by FTIR and SEM spectroscopy.

Related to the last important transition that was found, which is associated with the evaporation of water from the samples, it is found that samples *C* and *AF* are different (*p* < .05) compared with *IF*. This behavior is consistent with the fact that the *IF* samples have oil in their structure, which they acquired by immersion in the frying process, functioning as a pseudocoating, which modifies the thermal behavior in the water evaporation process, delaying the maximum temperature and increasing the energy requirements for it to happen (Ghaitaranpour et al., 2018).

The Cp (Figure 2b) reflects the structural changes that occur during the thermal analysis of the samples, corroborating that there are three important zones in the analysis: (1) melting of ice crystals; (2) gelatinization of the starch of the French fries, and (3) evaporation of water contained in the structures. In this sense, it is important to mention that melting and evaporation generate important structural rearrangements in French fries that have been subjected to immersion in oil, corresponding to what was obtained by SEM and texture analysis, where the crusts and surfaces change drastically (Touffet et al., 2020).

### 3.6 FTIR‐ATR analysis

**FIGURE 3:**
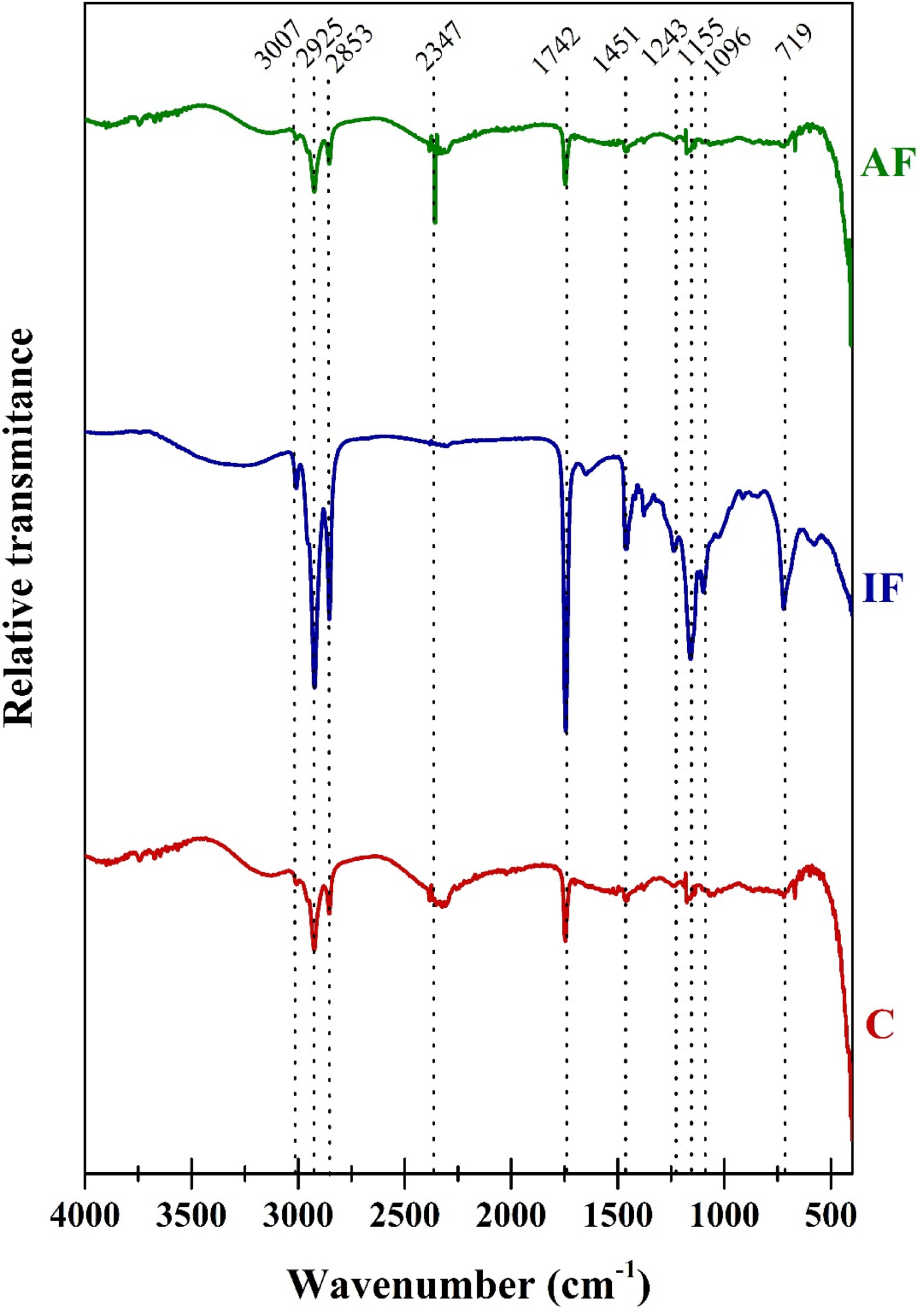
FTIR‐ATR spectra obtained for the samples analyzed at the different treatments from 4000 to 450 cm^−1^.

FTIR spectroscopy allows visualizing the vibrations of the functional groups and atoms, in this case, Figure 3 shows the results obtained among the samples analyzed. At 3007 cm^−1^ there is a marked band in the *IF* samples, which is found to a lesser extent in *C* and *AF*, this corresponds to C=C‐H stretching vibrations of the cis and/or trans fatty acids that are present mainly in canola oil used for deep frying. At 2925 cm^−1^ there are mainly stretching vibrations between C‐H that belong to potato starch; however, in the *IF* samples, this band is intensified because it is combined with the same vibrations that also come from canola oil (Li et al., 2022).

In this sense, it is important to mention that when these marked bands were found with greater intensity in the IF samples, it is directly attributed to the presence of canola oil in the French fries, since this is one of the main differentiating elements between the treatments, which is not present in samples *C* and *AF* submitted to spectroscopic analysis, which agrees with that obtained by MDSC.

At 2853 cm^−1^ there are mainly symmetric stretching vibrations of the methylene groups and C‐H tension vibrations of the aldehydes, in this same sense, it was found that the most intense band is found in the *IF* samples, unlike the one found in *C* and *AF* samples. At 2347 cm^−1^, a band was found only in *C* and *AF* samples, the latter being the one with the highest intensity. This band corresponds to stretching vibrations between C ≡ C of central carbons, this could be due to the formation of compounds by the interaction of hot air on the surface of the French fries (Manzoor et al., 2022).

In this case, it was found that the compounds formed in both samples do not belong to acrylamides or their derivatives, since according to what was reported by Li et al. (2022) and Manzoor et al. (2022) there are redox reactions between the ambient air and the surface of the French fries. That is why these reactions are accentuated when the air temperature in the frying chamber increases, unlike the *IF* samples where the oil acts as a protective barrier between the components of the environment and the surface of the analyzed sample.

In all the samples, another band appears at 1742 cm^−1^, which corresponds to stretching vibrations between C=O of the carboxylic acids and the aldehyde groups, coming from the starches, some traces of proteins and in the case of the *IF* samples which also come from canola oil for deep frying. Another band in the *IF* samples appears with great intensity at 1451 cm^−1^, which corresponds to the asymmetric torsion of ‐CH_3_ that derives mainly from the alkanic groups of the canola oil used to fry French fries (Jamwal et al., 2021). Pantalone et al. (2023) reported that this band also corresponds to the formation of acrylamides, indicating that the conventional frying process (*IF*) generates more of these undesirable compounds, unlike the air frying process.

Another band with great intensity in the *IF* samples appears at 1243 cm^−1^, which corresponds to tension vibrations in the C‐C(O)‐C groups of the acetates coming from the ester groups, which are intensified by the process of immersion in canola oil, unlike samples *C* and *AF*. At 1155 cm^−1^, tension vibrations were found in the S=O groups of the sulfones and R‐O‐R asymmetric stretching of the ether groups that come from the reactions between the starches and the oil, which is why in samples *C* and *AF*, the intensity of this band is minimal.

Another band was found at 1096 cm^−1^, which again predominates in the *IF* samples and corresponds to vibrations of asymmetric stretching between the C=O of the aliphatic ketones that are formed by the reactions that occurred during immersion frying. Finally, at 719 cm^−1^, there is a rolling torsion in the ‐CH_2_ of methylenes, mainly those formed by the reactions between potato starch and frying oil (Jamwal et al., 2021).

## 4 CONCLUSIONS

It was found that there are important differences between conventional immersion frying and air frying. The main difference lies in the use or not of oil due to the process; however, at a chemical, thermal, and structural level, air frying generates important changes on the surface, which are closely related to the textural and color. Studying the air frying process promotes novel alternatives in the production of products that have a lower fat content and, in turn, allow diversifying the options that are currently on the market, maintaining and in some cases improving the main physicochemical characteristics of French fries which in turn influence the purchase decision of consumers.

## AUTHOR CONTRIBUTIONS

**Jonathan Coria‐Hernández:** Conceptualization (equal); data curation (equal); formal analysis (equal); methodology (equal); writing – original draft (equal); writing – review and editing (equal). **José Luis Arjona‐Román:** Conceptualization (equal); funding acquisition (equal); investigation (equal); project administration (equal); supervision (equal). **Rosalía Meléndez‐Pérez:** Data curation (equal); formal analysis (equal); project administration (equal); supervision (equal); validation (equal); writing – review and editing (equal).

## CONFLICT OF INTEREST STATEMENT

The authors declare that they have no conflict of interest.

## ETHICAL APPROVAL

This article does not contain any studies with human participants or animals performed by authors.

## CONSENT TO PARTICIPANT

All authors read the manuscript and gave final approval for publication.

## Data Availability

The datasets used and/or analyzed during this study are available from the corresponding author upon reasonable request.
